# Predictors of frequency of 1-year readmission in adult patients with diabetes

**DOI:** 10.1038/s41598-023-47339-7

**Published:** 2023-12-16

**Authors:** Jade Gek Sang Soh, Amartya Mukhopadhyay, Bhuvaneshwari Mohankumar, Swee Chye Quek, Bee Choo Tai

**Affiliations:** 1 Saw Swee Hock School of Public Health, National University of Singapore, Singapore, Singapore; 2 Health and Social Sciences, Singapore Institute of Technology, Singapore, Singapore; 3 Respiratory and Critical Care Medicine, National University Hospital, Singapore, Singapore; 4 Yong Loo Lin School of Medicine National University of Singapore, Singapore, Singapore; 5 Medical Affairs, Alexandra Hospital, Singapore, Singapore; 6 Medical Affairs – Clinical Governance, National University Hospital, Singapore, Singapore; 7 Department of Pediatric Cardiology, National University Hospital, Singapore, Singapore

**Keywords:** Diseases, Endocrinology, Health care, Risk factors

## Abstract

Diabetes mellitus (DM) is the third most common chronic condition associated with frequent hospital readmissions. Predictors of the number of readmissions within 1 year among patients with DM are less often studied compared with those of 30-day readmission. This study aims to identify predictors of number of readmissions within 1 year amongst adult patients with DM and compare different count regression models with respect to model fit. Data from 2008 to 2015 were extracted from the electronic medical records of the National University Hospital, Singapore. Inpatients aged ≥ 18 years at the time of index admission with a hospital stay > 24 h and survived until discharge were included. The zero-inflated negative binomial (ZINB) model was fitted and compared with three other count models (Poisson, zero-inflated Poisson and negative binomial) in terms of predicted probabilities, misclassification proportions and model fit. Adjusted for other variables in the model, the expected number of readmissions was 1.42 (95% confidence interval [CI] 1.07 to 1.90) for peripheral vascular disease, 1.60 (95% CI 1.34 to 1.92) for renal disease and 2.37 (95% CI 1.67 to 3.35) for Singapore residency. Number of emergency visits, number of drugs and age were other significant predictors, with length of stay fitted as a zero-inflated component. Model comparisons suggested that ZINB provides better prediction than the other three count models. The ZINB model identified five patient characteristics and two comorbidities associated with number of readmissions. It outperformed other count regression models but should be validated before clinical adoption.

## Introduction

Worldwide, the estimated number of adults with diabetes mellitus (DM) is projected to increase to nearly 700 million in 2045 from 451 million in 2017^1^. Although pharmacological interventions, self-management education and lifestyle modification can help to achieve optimal glycaemic control and improve clinical outcomes^2^, unplanned hospital readmission in adult patients with DM remains high^3,4^. Globally, the 1-year readmission rate among patients with DM ranges from 25.1 to 29.7%^5–7^ making DM the third most common chronic condition associated with frequent readmission^7^. In 2015, the average cost per patient with three or more hospitalisations within 1 year in Singapore was approximately USD$22,000 which is 10 times more than the average cost of a patient with no readmission^8^. It is anticipated that readmission among patients with DM maybe more than one episode within 1 year^9^ and each episode is costly. It is also known that patients with DM consume more healthcare resources than those without^10^. Identifying the predictors associated with number of readmission within 1-year among patients with diabetes may allow healthcare providers to mitigate the number of readmissions by targeting at modifiable risk factors, thereby resulting in cost savings^8^. However, the number of readmissions within 1 year among patients with DM is less often studied^9^ as compared to 30-day readmission in which independent risk factors^11,12^ and prediction models^4,13–15^ have been identified. Predictors of the number of readmissions in 1 year among patients with DM may be different from predictors for readmission within 30 days.

The number of readmissions within 1 year may not be associated with the quality of care received during index admission but may be a reflection of disease progression^16,17^. This argument is particularly relevant where the index admission was DM-related; this group of patients may persistently fail to maintain good glycaemic control and have frequent readmission^18^. Current literature^7,8,19^ suggests that the frequency of readmission within 1 year is conventionally classified as a binary outcome such as frequent readmission (i.e., one index admission and at least two readmissions) versus non-frequent readmission. However, another study has defined it as having two or more hospitalisations in a year^20^ which may lead to estimation bias^21^. Dichotomising the actual number of readmissions as a binary variable may lead to a false negative (type 2 error) study conclusion^22^. In addition, the number of readmissions is a count variable, and tends to have excessive zeroes and its distribution may be overdispersed, that is, its variance may be relatively larger than its mean^23,24^. Thus, for such outcomes, regression models for count variables with zero-inflated and overdispersed distributions may be more appropriate than a binary logistic regression model in identifying predictors of number of readmission within 1-year. In count regression models, the non-admissions (count zero) are modelled separately from the number of admissions (non-zero count) to accurately predict the number of readmissions without the influence of non-admission.

### Study objective

The primary objective of this study is to identify the independent risk factors associated with number of hospital readmissions within 1 year amongst adult patients with DM whose index diagnosis was DM-related; we took into account the possibility of excessive zeroes as well as overdispersion which are recorded for the count outcome. A secondary objective is to compare this primary model with other multivariable count regression models that do not account for zero-inflation or overdispersion, with respect to model fit.

## Methodology

**Figure 1:**
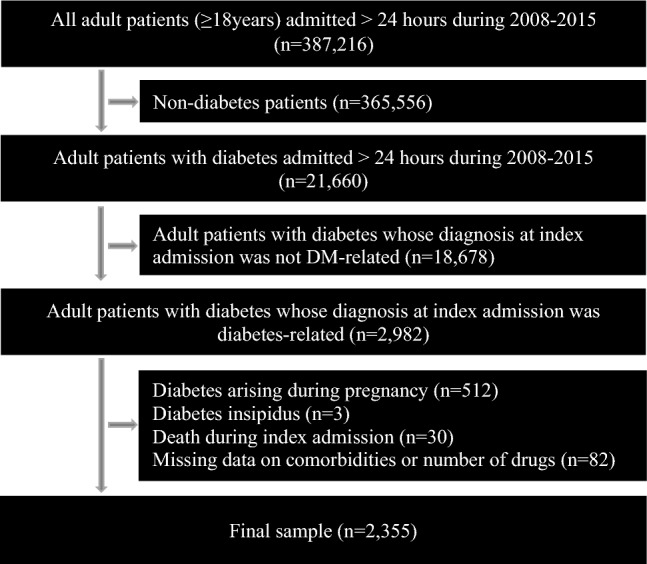
Determination of study cohort.

Electronic medical records from the National University Hospital (NUH), Singapore were extracted retrospectively from January 2008 to December 2015 from various linked hospital databases. Index admission was defined as the first admission during the study period. The study population consisted of patients who were 18 years or older at the time of the index admission who had a hospital stay of more than 24 h. Their primary or secondary diagnoses at index admission were DM-related based on the International Classification of Diseases, Ninth Revision, Clinical Modification (ICD-9-CM from 2007 to 2011) and the International Classification of Diseases-Tenth Revision (ICD-10AM, 2012 onwards). These encompassed but were not limited to, diabetic ketoacidosis, hypoglycaemia, impaired glucose regulation, abnormal glucose tolerance with previous history of DM, elevated blood glucose level with previous history of DM and pre-existing DM in pregnancy. Exclusions were DM during pregnancy, diabetes insipidus, death during index admission and missing data on comorbidities number of drugs administered. There were 2355 patients included in the analysis after applying the criteria (Fig. 1).

### Dependent variable

The dependent variable was the number of readmissions within 1 year after an index admission. Index hospitalisation was defined as first admission during the study period, with the exclusion of patients whose “first admission” was actually a readmission within the previous 30 days at the start of the study. For example, if a patient had been admitted on both 15 December 2007 and 1 January 2008, the second admission would be excluded as a case of index admission.

### Independent variables

Demographic characteristics included gender, age, race and residency status. Past medical history including comorbidities, number of surgical procedures, emergency department (ED) visits in the 12 months prior to the index admission and number of drugs at point of discharge. Admission and discharge information included hospital length of stay (LOS), LOS in Intensive Care Unit, classification of index hospital admission (emergency or elective), hospital ward subsidy (a proxy measurement of socioeconomic status) and discharge destination (e.g., home or step-down care facilities). Other relevant medical data included the Charlson Comorbidity Index^25^ (CCI) and its listed comorbidities. However, CCI was not considered in the regression analysis as it was derived from the individual comorbidities, with greater weightage allocated to more severe conditions such as AIDS and metastatic tumour; these are not known predictors of frequency of readmission within 1 year amongst patients with DM, although they are predictors of 10-year survival^25^.

### Statistical analysis

For simplicity in reporting the baseline characteristics, the number of readmissions was categorised into four groups (0, 1, 2, ≥ 3 readmissions) and the proportion readmitted within a year was compared for each characteristic or comorbidity using a Chi-square or Fisher’s exact tests as appropriate. For continuous variables such as age, number of drugs, LOS, and CCI which were not normally distributed, the Kruskal–Wallis test was used. The dependent variable was assessed graphically for excessive zeros in the observed frequencies. Its variance was checked to see if it was higher than the mean to determine whether there was overdispersion. Associations between the number of readmissions within 1 year and the risk factors were first analysed using zero-inflated negative binomial (ZINB) regression as the primary model, to account for possible overdispersion and excessive zeroes with respect to the count outcome. The ZINB model assumes that the outcome is modelled to two different processes. Presence of excessive zeroes for number of readmission was modelled using logistic regression. The expected number of readmissions was modelled using a negative binomial model. Each risk factor was first individually assessed for significance in the respective components of the ZINB model. Only the significant variables associated with each component were included in the initial multivariable ZINB model, after considering possible collinearity. Thus, although LOS, age and number of drugs were significant in the bivariate analysis of both the logistic and negative binomial models, the zero-inflated components for age and number of drugs were no longer significant after adjustment for other variables. In addition, the predicted probabilities of readmissions within 1 year were plotted against the corresponding observed probabilities; if the predicted probability corresponded closely to the observed, the model was considered a good fit. The model was further assessed by estimating the proportion misclassified.

Three other count models, namely the Poisson regression model (PRM), zero-inflated Poisson (ZIP) regression model and negative binomial regression model (NBRM), were considered for comparison with ZINB. The model building approach as described for ZINB was adopted for each of these models. A Vuong test was used to compare ZINB with NBRM, where statistical significance suggested the inclusion of the zero-inflation parameter fitted better than a negative binomial model^26^. Similarly, the Vuong test was also used to compare and ZIP with PRM. A likelihood ratio test of alpha = 0 was conducted to assess overdispersion, where a significant test implies evidence of overdispersion. Model comparisons were also assessed by the Akaike information criterion (AIC) and the Bayesian information criterion (BIC), where a lower value for either criterion implies a better fit.

The level of significance was set at 0.05 assuming a two-sided test. All statistical analyses were conducted using Stata version 16 (STATA Corp, College Station, Texas, USA).

### Institutional review board approval

This retrospective study was approved by the National Healthcare Group Domain Specific Review Board (Reference no: 2016/00339) with waiver of informed consent obtained. All study methods were conducted in accordance with the recommended guidelines and regulations.

## Results

### Description of patient characteristics

**Figure 2:**
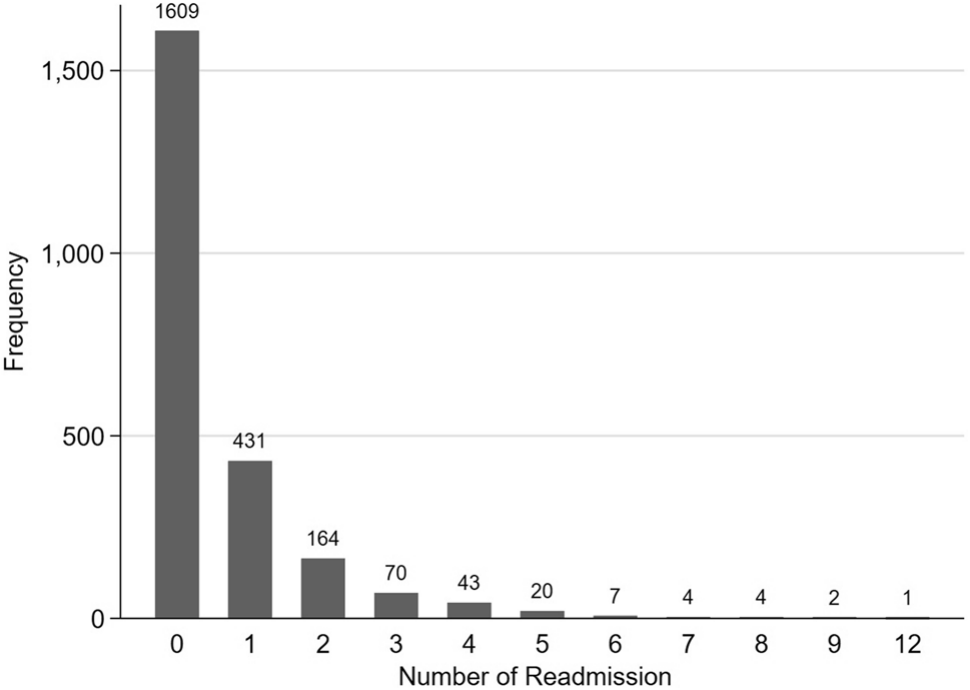
Frequency of number of readmissions in a year.

Demographic characteristics of the study cohort involving 2355 patients were summarised according to number of readmissions within 1 year (Table 1). Overall, 31.7% of the patients had at least one admission after the index hospitalisation. The admission rates for 1, 2 and 3 or more readmissions were 18.3%, 7.0% and 6.4% respectively. There were slightly more males (52.6%) in the study cohort, but gender was not significantly associated with number of readmissions. Those with at least 1 readmission were significantly older than those without readmission. The proportions of Singapore residents who were readmitted increased with the number of readmissions [no readmission: 1429 (88.8%), 1 readmission: 406 (94.2%), 2 readmissions: 160 (97.6%) and 3 or more readmissions: 146 (96.7%)]. Similar trend was observed for having one or more surgical operations. Number of drugs, LOS and type of ward accommodation (private versus subsidised) were positively associated with number of readmissions without any definite trend. Five comorbidities (diabetes chronic complication, renal disease, peripheral vascular disease [PVD], ischaemic heart disease [IHD] and peptic ulcer disease) were identified as risk factors for number of readmissions within a year among patients with DM in the bivariate analysis (Table 2). Figure 2 summarises the number of readmissions in a year; zero readmissions had by far the highest frequency, suggesting the variable was zero-inflated and possibly overdispersed since the variance was relatively larger than the mean.

**Table 1:** Characteristics of 2355 participants by number of readmissions within 1-year.

Demographic characteristic	Total (n = 2355, 100%)	Number of readmissions				P-value
		0 (n = 1609, 68.3%)	1 (n = 431, 18.3%)	2 (n = 164, 7.0%)	> 3 (n = 151, 6.4%)	
Gender						0.063
Female	1116 (47.4)	757 (47.1)	223 (51.7)	65 (39.6)	71(47.0)	
Male	1239 (52.6)	852 (52.9)	208 (48.3)	99 (60.4)	80 (53.0)	
Age in years, median (IQR)^^^	59 (48,69)	57 (46,67)	61 (49,72)	60, (51,70)	62 (53,72)	** < 0.001**
Ethnicity						0.582
Chinese	1171 (49.7)	788 (49.0)	217 (50.3)	84 (51.2)	82 (54.3)	
Malay	601 (25.5)	408 (25.4)	115 (26.7)	42 (25.6)	36 (23.9)	
Indian	362 (15.4)	245 (15.2)	68 (15.8)	26 (15.9)	23 (15.2)	
Others	221 (9.4)	168 (10.4)	31 (7.2)	12 (7.3)	10 (6.6)	
Residential status^^^						< 0.001*
Non-resident	214 (9.1)	180 (11.2)	25 (5.8)	4 (2.4)	5 (3.3)	
Singapore resident	2141 (90.9)	1429 (88.8)	406 (94.2)	160 (97.6)	146 (96.7)	
Medical history						
No. of surgical operations						** < 0.001**
0	1755 (74.5)	1225 (76.1)	308 (71.5)	117 (71.3)	105 (69.5)	
> = 1	600 (25.5)	384 (23.9)	123 (28.5)	47 (28.7)	46 (30.5)	
No. of emergency department visits in the past 12 months^^^	1(1,1)	1(1,1)	1(1,1)	1(1,1)	1(1,1)	** < 0.001**
Number of drugs, median (IQR)^^^	8 (5,11)	8 (5,11)	9 (6,12)	10 (7,13)	10 (8,14)	** < 0.001**
Admission and discharge information						
Length of stay (days) (IQR)^^^	4 (2,7)	3 (2,6)	4 (2,7)	5 (3,9)	5 (3,12)	** < 0.001**
ICU length of stay						0.255
0	2255 (95.7)	1546 (96.1)	414 (96.1)	153 (93.3)	142 (94.0)	
> = 1	100 (4.3)	63 (3.9)	17 (3.9)	11 (6.7)	9 (6.0)	
Type of hospital admission						0.374
Non-emergency	149 (6.3)	100 (6.2)	34 (7.9)	8 (4.9)	7 (4.6)	
Emergency	2206 (93.7)	1509 (93.8)	397 (92.1)	156 (95.1)	144 (95.4)	
Type of ward accommodation						** < 0.001**
Private	357 (15.2)	280 (17.4)	56 (13.0)	8 (4.9)	13 (8.6)	
Subsidised	1998 (84.8)	1329 (82.6)	375 (87.0)	156 (95.1)	138 (91.4)	
Discharge type						0.757
Discharged home/discharged to home with day rehab or medical appointment	2225 (94.5)	1524 (94.7)	407 (94.4)	154 (93.9)	140 (92.7)	
Discharged to other hospitals or nursing homes/discharged against medical advice/absconded	130 (5.5)	85 (5.3)	24 (5.6)	10 (6.1)	11 (7.3)	

Unless otherwise indicated, cells show number (percentage).

Significant values are in bold.

*IQR* interquartile range, *ICU* intensive care unit.

*Fisher exact test.

^^^Variable included in the final model.

**Table 2:** Comorbidities of participants by number of readmissions within 1-year.

Comorbidity	Total (n = 2355, 100%)	Number of readmissions				P-value
		0 (n = 1609, 68.3%)	1 (n = 431, 18.3%)	2 (n = 164, 7.0%)	> 3 (n = 151, 6.4%)	
CCI, median (IQR)	3 (1,4)	3 (1,4)	3 (2,4)	3 (3,5)	3 (2,5)	** < 0.001**
Diabetes chronic complication	1582 (67.2)	1030 (64.0)	299 (69.4)	136 (82.9)	117 (77.5)	** < 0.001**
Renal disease^^^	550 (23.4)	317 (19.7)	111 (25.8)	57 (34.8)	65 (43.1)	** < 0.001**
Heart failure	162 (6.9)	111 (6.9)	25 (5.8)	17 (10.4)	9 (6.0)	0.251
Peripheral vascular disease^^^	142 (6.0)	72 (4.5)	37 (8.6)	20 (12.2)	13 (8.6)	** < 0.001**
Ischaemic heart disease	97 (4.1)	49 (3.1)	24 (5.6)	12 (7.3)	12 (8.0)	**0.001**
Liver disease	58 (2.5)	41 (2.5)	7 (1.6)	2 (1.2)	8 (5.3)	0.086*
Dementia	34 (1.4)	22 (1.4)	11 (2.5)	1 (0.6)	0 (0.0)	0.098*
Chronic obstructive pulmonary disease	21 (0.9)	13 (0.8)	6 (1.4)	2 (1.2)	0 (0.0)	0.381*
Peptic ulcer disease	21 (0.9)	7 (0.4)	9 (2.1)	4 (2.4)	1 (0.7)	0.002*
Any tumour	19 (0.8)	11 (0.7)	4 (0.9)	2 (1.2)	2 (1.3)	0.467*
Cerebrovascular disease hemiplaegia	12 (0.5)	11 (0.7)	0 (0.0)	1 (0.6)	0 (0.0)	0.254*
Metastatic tumour	6 (0.3)	4 (0.2)	0 (0.0)	2 (1.2)	0 (0.0)	0.143*
Connective tissue disease	5 (0.2)	2 (0.1)	2 (0.5)	0 (0.0)	1 (0.7)	0.210*
Acquired immune deficiency syndrome	2 (0.1)	1 (0.1)	0 (0.0)	1 (0.6)	0 (0.0)	0.283*

Unless otherwise indicated, cells show number (percentage).

Significant values are in bold.

*CCI* Charlson comorbidity index, *IQR* interquartile range.

*Fisher’s exact test.

^^^Variable included in the final model.

### ZINB model in predicting the number of readmissions in a year

The final multivariable ZINB prediction model included seven significant predictors (PERUSAL): **P**VD, number of **E**D visits, **R**enal disease, number of dr**U**gs, residential **S**tatus, **A**ge and zero-inflated **L**OS (Table 3). The expected number of readmissions amongst those with PVD was 1.42 times (95% confidence interval (CI): 1.07, 1.90) compared to those without PVD. There was a 27% increase (95% CI 1.15, 1.41) in the expected number of readmissions for every additional visit to ED. The expected number of readmissions amongst those with renal disease was 1.60 times (95% CI 1.34, 1.92) compared to those without renal disease. A 5% increase (95% CI 1.03, 1.07) in the expected number of readmissions for every additional drug was noted. The expected number of readmissions amongst Singapore residents was 2.37 times (95% CI 1.67, 3.35) compared to non-residents. There was a 1% increase (95% CI 1.001, 1.012) in the expected number of readmissions for every additional year in age. For every one-day increase in LOS, the odds of no readmissions were multiplied by a factor of 0.64 (95% CI 0.44, 0.92). Equivalently, after taking its reciprocal, the odds of one or more readmissions were increased by a factor of 1.56 (95% CI 1.09, 2.27).

**Table 3:** Predictors of number of hospital readmissions within 1 year in the bivariate and multivariable analyses (n = 2355).

	Bivariate analysis				Multivariable PERUSAL model^^^		
Predictor	Exp(*β*)*	95% CI	P-value	Exp(*β*)*		95% CI	P-value
Peripheral vascular disease	1.67	(1.23, 2.26)	0.001	1.42		(1.07, 1.90)	0.016
ED visit	1.27	(1.14, 1.42)	< 0.001	1.27		(1.15, 1.41)	< 0.001
Renal disease	1.95	(1.64, 2.31)	< 0.001	1.60		(1.34, 1.92)	< 0.001
Number of drugs	1.09	(1.07, 1.11)	< 0.001	1.05		(1.03,1.07)	< 0.001
Residential status: resident	2.70	(1.91, 3.83)	< 0.001	2.37		(1.67, 3.35)	< 0.001
Age (years)	1.01	(1.010, 1.020)	< 0.001	1.01		(1.001, 1.012)	0.017
Zero-inflated variable: length of stay	0.70	(0.58, 0.85)	< 0.001	0.64		(0.44, 0.92)	0.015

*β refers to difference in log of expected count for binary risk factors or increase in log of expected count per unit increase in continuous risk factor respectively. For the zero-inflated parameter, it refers to increase in log odds ratio per unit increase in the zero-inflated LOS variable.

^^^The initial multivariable also included: LOS, type of ward accommodation, diabetes chronic complication, IHD, peptic ulcer disease, zero-inflated age and zero-inflated number of drugs.

### Comparison of ZINB with other count models

The estimated regression coefficients of PRM and NBRM for all the independent variables were similar to those of the non-zero-inflated predictors of ZINB (Table 4). ZIP yielded numerically smaller regression coefficients for all the predictors as compared to ZINB and the other two count regression models. Zero-inflated LOS was not a significant predictor in ZIP but was significant in ZINB (Table 4). Similarly, LOS was not a significant predictor in the other two non-zero-inflated count models, PRM and NBRM.

**Table 4:** Estimated regression coefficients and the 95% CIs for the four prediction models.

Predictor	ZINB	ZIP	NBRM	PRM
Peripheral vascular disease	0.353 (0.067, 0.639)	0.224 (0.020, 0.427)	0.423 (0.129, 0.718)	0.396 (0.213, 0.579)
Emergency department visit	0.239 (0.138, 0.341)	0.180 (0.117, 0.243)	0.245 (0.142, 0.348)	0.231 (0.174, 0.288)
Renal disease	0.472 (0.293, 0.650)	0.426 (0.285, 0.567)	0.463 (0.283, 0.644)	0.481 (0.363, 0.600)
Number of drugs	0.045 (0.025, 0.065)	0.042 (0.027, 0.057)	0.055 (0.036, 0.074)	0.049 (0.037, 0.062)
Residential status: resident	0.862 (0.516, 1.210)	0.829 (0.505, 1.152)	0.863 (0.515, 1.211)	0.871 (0.584, 1.158)
Age (year)	0.006 (0.001, 0.012)	0.005 (0.001, 0.010)	0.006 (0.001, 0.012)	0.006(0.002, 0.010)
Zero-inflated variable: length of stay (day)	− 0.449 (− 0.810, − 0.087)	− 0.008 (− 0.019, 0.002)	–	–
Tests and fit statistics				
Vuong test statistic	1.57^a^	7.30^b^	–	–
P-value	0.058	< 0.001		
Likelihood-ratio statistic for test of alpha = 0	133.41	–	496.42	–
P-value	< 0.001		< 0.001	
AIC	4661.382	4792.795	4667.561	5161.98
BIC	4719.025	4844.674	4713.676	5202.33

*ZINB* zero-inflated negative binomial regression model, *PRM* Poisson regression model, *ZIP* zero-inflated Poisson regression model, *NBRM* negative binomial regression model, *AIC* Akaike information criterion, *BIC* Bayesian information criterion.

^a^Vuong test statistic of ZINB versus NBRM.

^b^Vuong test statistic of ZIP versus PRM.

The Vuong test of ZINB versus NBRM (Table 4) suggested weak evidence of zero-inflation (p = 0.058) although it was more clearly demonstrated in the comparison of ZIP versus PRM (p < 0.001). The likelihood-ratio test for alpha = 0 was significant (χ^2^ = 133.41, df = 1, p < 0.001), suggesting overdispersion and thus indicating ZINB was preferred to ZIP (Table 4). The same test also indicated that NBRM was preferred to PRM (χ^2^ = 496.42, df = 1, p < 0.001). The ZINB model had the lowest AIC value while the NBRM model had the lowest BIC value (Table 4). The differences in AIC and BIC between ZINB and NBRM models were, however, marginal.

**Figure 3:**
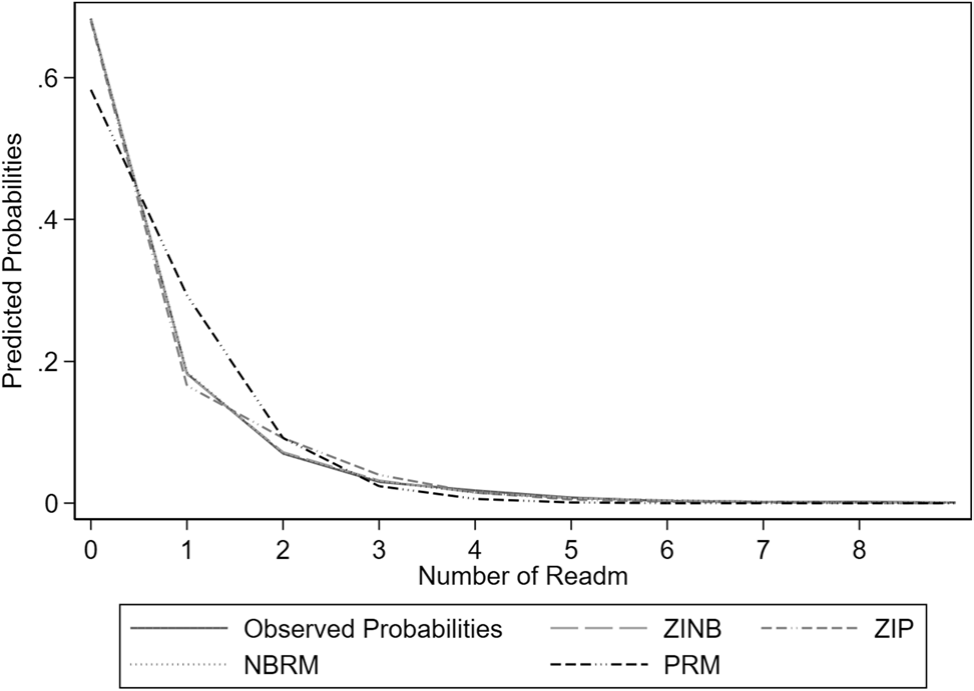
Comparing predicted probabilities of number of readmissions in a year between observed probabilities versus other count models. *ZINB* zero-inflated negative binomial regression model, *ZIP* zero-inflated Poisson regression model, *NBRM* negative binomial regression model, *PRM* Poisson regression model.

Figure 3 shows the predicted probabilities of ZINB versus the other three count data regression models (ZIP, PRM and NBRM). Compared to ZINB, ZIP underestimated the probability of one readmission but overestimated the probabilities for two and three readmissions. Conversely, PRM overestimated the probabilities for one and two readmissions and grossly underestimated the probably of zero readmission. Both ZINB and NBRM prediction models appeared to provide equally good fit, with lines largely overlapping and almost indistinguishable.

Based on the differences between the observed and the predicted number of readmissions estimated from each model (Table 5), the percentages of misclassification for ZINB, PRM, ZIP, and NBRM were 1. 4%, 25.8%, 6.4% and 1.2%, respectively. The results suggest ZINB and NBRM were able to predict more accurately than the other two count models. The slight difference between NBRM and ZINB in terms of the percentage of misclassification appeared to be negligible. However, ZINB (18.2%) provided better prediction than NBRM (18.5%) for patients who had one readmission in 1 year (18.3%). On the other hand, NBRM (3.1%) provided slightly better prediction than ZINB (3.2%) for patients who had 3 readmissions in the same period (3.0%). Both models yielded the same predicted probabilities for number of readmissions ≥ 4.

**Table 5:** Observed and predicted number of readmissions within 1 year.

Number readmission	Observed N (%)	Predict N (%)			
		ZINB	PRM	ZIP	NBRM
0	1609 (68.3)	1611 (68.4)	1373 (58.3)	1601 (68.0)	1608 (68.3)
1	431 (18.3)	429 (18.2)	690 (29.3)	391 (16.6)	436(18.5)
2	164 (6.9)	170 (7.2)	217 (9.2)	217 (9.2)	165 (7.0)
3	70 (3.0)	75 (3.2)	57 (2.4)	94 (4.0)	73 (3.1)
4	43 (1.8)	35 (1.5)	14 (0.6)	35 (1.5)	35 (1.5)
5	20 (0.8)	16 (0.7)	2 (0.1)	12 (0.5)	16 (0.7)
6	7(0.3)	9 (0.4)	0 (0)	5 (0.2)	9 (0.4)
7	4 (0.2)	5 (0.2)	0 (0)	2 (0.1)	5 (0.2)
8	4 (0.2)	2 (0.1)	0 (0)	0 (0)	2 (0.1)
9	2 (0.1)	2 (0.1)	0 (0)	0 (0)	2 (0.1)
12	1 (0.04)	–	–	–	–
Number misclassified (%)	–	32 (1.4)	608 (25.8)	151 (6.4)	27 (1.2)

## Discussion

This study explored a longer timeframe because an increase in the number of readmissions within 1 year after an index admission in patients with DM is common^7,8^. In comparison with our previous study^27^, LOS, number of drugs and PVD were common predictors of 30-day readmission and number of 1-year readmissions whereas IHD was a predictor of the former but not the latter. In addition, age, number of ED visits, residential status and renal disease were not previously reported as predictors of 30-day readmission from the same study population^27^. Studies which studied 1-year readmission amongst patients with DM aimed to evaluate the outcome associated with a specific treatment^28,29^. Thus the objectives of such published papers^28,29^ were different from this study.

Our prevalence rate for one or more readmissions within the first year following an index admission was 31.7%. This was higher than a study^6^ which specifically focused on diabetic foot problems (25.1%) but similar to another involving patients who had diabetic ketoacidosis (29.7%)^5^. The figures suggest that readmission within 1 year is at least 1 in 4 among patients with DM^5^. The PERUSAL model identified five patient characteristics (residential status, age, ED visits, LOS and number of drugs) and two comorbidities (PVD and renal disease) as the predictors of number readmission within 1 year among patients with a DM-related diagnosis at index admission.

### Patient characteristics

Being a Singapore resident was associated with a higher expected number of readmissions within 1 year as compared with non-residents. Although healthcare is not free to all residents, a substantial amount of inpatient healthcare cost is subsidised^30^. In addition, out-of-pocket payments for hospital treatment are relatively lower compared to other health services such as community care services^30^. Thus, inpatient treatment cost is not a barrier to deter a majority of Singapore residents from unplanned readmissions. Similarly, it was found that readmission was higher among Medicare, Medicaid and commercially insured patients as compared to patients who paid for their own care^31^. The percentage of non-residents in Singapore was about 25% in 2010^32^ which suggested that the proportion readmitted (9.1%) underrepresented this population. Higher treatment costs and returning to their country of origin, perhaps for treatment, could explain the underrepresentation. In general, foreign patients were more likely to be admitted for trauma related injuries and defaulted non-trauma conditions treatment^33^. Comparison with another similar local study^7^ was not possible because it had excluded non-residents from the analysis. Nevertheless, the prevalence of diabetes (9.1%) among non-residents may be relevant in terms to the government agency responsible for recruiting foreign workers to work in Singapore. Increase in age was associated with increase in number of readmissions within a year among patients with DM. Age was also a predictor in a study which determined all-cause readmission within a year following a surgical procedure among older adults with diabetes^29^. Number of ED visits prior to index readmission was found to be associated with frequent readmission within 1 year among older patients^7^ while DM-related symptoms and DM treatments were risk factors for ED attendance in another study^34^. If admitted, patients with DM were also found to have longer LOS as compared with those without^35^. A longer LOS during index admission could be related to complications^36^ and serve as an indicator of disease burden^37^. LOS was also found to be independently associated with 30-day readmission^12,14,38^ and frequent readmission within a year among the adult population^39^. Polypharmacy is defined as having more than five medicines^40^ and it was an independent predictor of unplanned hospital readmission at 1-year among patients with critical illness^41^. Other than receiving anti-diabetic drugs, most patients would also be prescribed with cardioprotective medication, and the number of drugs also increased over time after diagnosis^42^. For example, in a study by Black et al.^42^ the median number of medications increased from 2 at diagnosis to 6 five years later. Polypharmacy has been shown to be associated with drug-drug interaction and hospitalisation among older adults with DM^43^. In addition, medication nonadherence associated with side effects^44^ could be another reason in explaining why number of drugs was associated with readmission at 1 year among patients with DM^37^. The above patient characteristics suggest that older adults with DM with a longer LOS would be more likely to have one or more readmissions within 1 year. They may also utilise more healthcare resources with higher ED visits and be prescribed more medications. Therefore, planning for healthcare infrastructure and services which involve manpower and cost should include the health needs of older adults with DM.

### Comorbidities

PVD was more common in patients with DM as compared to those without DM^45^. Renal disease was also a common complication of DM^46^ and an independent predictor of unplanned readmission within 1 year^37^. Although some comorbidities are not modifiable risk factors, good glycaemic control is still necessary to delay the progression of renal disease and PVD^47^. Healthcare professionals should strive to develop innovative tertiary prevention programmes to ensure medication adherence and adequate glycaemic control for patients with DM to prevent unplanned hospital readmission. This may translate to a reduction in healthcare cost of treating DM-related complications and procedures such as renal dialysis or limb amputation which have physiological and psychological detrimental effects on the patients.

### Model accuracy

In terms of model accuracy, PRM was the least accurate model as there was suggestion of overdispersion in the readmission data. ZIP did not account for overdispersion and thus it was not as accurate as ZINB. The predicted probabilities of number of readmissions of ZINB were similar to those of NBRM. LOS was not a significant predictor in the NBRM model, but the zero-inflated LOS was significant in the ZINB model. In this respect, ZINB may be a more appropriate prediction model for the number of readmissions within 1 year among patients with DM because LOS has been shown to be an important predictor of frequent hospital readmission within a year^39^. However, the Vuong test (p = 0.058) did not suggest that ZINB fitted appreciably better than NBRM. A p-value on its own provides limited information to clinical relevance^48^. Thus model selection should not be solely based on statistical inference but also on evidence of other relevant study findings^48^.

Ordinal logistic regression is sometimes implemented for the analysis of count data^49^. This model is quantified based on log OR whereas our secondary objective is to compare count regression models in predicting number of readmissions. In addition, the number of ordinal categories may affect the study result in terms of its strength of association with the predictors^50^. The ordinal cut points for the outcome of interest are often arbitrarily chosen and may not be sensitive to detect associations^50^. Interestingly, ordinal logistic regression identified IHD as a significant predictor although it was not a significant predictor in any of the count regression models we considered. It also failed to detect LOS as a significant predictor (Appendix A). Its percentage of misclassification was 2.6% when number of re-admissions > 4 within 1 year was combined into a single category.

### Study strengths and limitations

A strength of this study is that the number of unplanned hospital readmissions within 1 year was analysed using count regression models, and the respective models were compared prior to recommending the most appropriate one for reporting. The study findings may be generalised to other public hospitals in developed countries where determinants of health are not differentiated by geographical location^51^. The PERUSAL model is also relevant in clinical practice for identifying at-risk patients because the data are readily available in databases of the hospitals and can be automated in modern electronic health records. However, some study limitations should also be noted. The data only included patients readmitted to NUH and we had no access to data from other hospitals if the study patients had sought treatment in another hospital after the index admissions. In addition, we did not have mortality information after index hospital discharge, so we were unable to exclude individuals who had died within 1 year of discharge. Such individuals were likely to have a higher prevalence of risk factors for readmission but would not have been readmitted due to their deaths, potentially leading to underestimation of the risk factors. The secondary database did not include other important predictors of readmission such as the glycated haemoglobin level, duration of DM, specific DM medications and mental health status^4,11^. Although the model was based on a consecutive inpatient sample over a span of 7 years, it was not formally validated in a larger prospective study. Thus, further research may be warranted to validate the PERUSAL model in prospective cohort studies before adapting it for implementation in practice. Healthcare subvention and payment methods vary in different countries which should also be taken in the consideration.

## Conclusion

A prediction model, PERUSAL, demonstrated that the number of readmissions within a year readmission amongst patients with DM whose index diagnosis was DM-related was associated with age, residential status, zero-inflated LOS, number of ED visits, number of drugs administered, PVD and renal disease. A ZINB model was preferred over other relevant count model regressions because it accounted for overdispersed and zero-inflated outcome data.

### Supplementary Information

Supplementary Information.

## Data Availability

The datasets used and analysed during the current study are available from the corresponding author on reasonable request.
