# Supplementary material for: Predictors of frequency of 1-year readmission in adult patients with diabetes
Source: Sci Rep. 2023 Dec 16;13:22389. doi: 10.1038/s41598-023-47339-7 (PMC10725424; doi:10.1038/s41598-023-47339-7)
Supplement: Supplementary file 1 — Supplementary Information. [file 41598_2023_47339_MOESM1_ESM.pdf]

Appendix A: Estimated regression coefficients of ordinal logistic regression model

Estimated regression coefficients of ordinal model with 95% CIs	
**Comorbidity**	**β**
**Peripheral vascular disease**	0.619 (0.288, 0.951)
**Emergency department visit**	0.303 (0.184, 0.421)
**Renal disease**	0.476 (0.268, 0.685)
**Number of drugs**	0.053 (0.031, 0.075)
**Residential status: resident**	0.906 (0.518, 1.294)
**Age (years)**	0.007 (0.001, 0.013)
**Ischaemic heart disease**	0.463 (0.062, 0.863)
The coefficient β is the log-odds of readmission, where e^{β} is the odds ratio of readmission.	
